# SARS-CoV-2 spike antibody concentration in gamma globulin products from high-prevalence COVID-19 countries are transmitted to X-linked agammaglobulinemia patients

**DOI:** 10.3389/fimmu.2023.1156823

**Published:** 2023-03-29

**Authors:** Allon Raphael, Oded Shamriz, Ariella Tvito, Sophie Magen, Shmuel Goldberg, Orli Megged, Atar Lev, Amos J. Simon, Yuval Tal, Raz Somech, Rachel Eisenberg, Ori Toker

**Affiliations:** ^1^ Pediatric Department, Shaare Zedek Medical Center, Jerusalem, Israel; ^2^ Allergy and Clinical Immunology Unit, Department of Medicine, Hadassah Medical Organization, Faculty of Medicine, Hebrew University of Jerusalem, Jerusalem, Israel; ^3^ The Lautenberg Center for Immunology and Cancer Research, Institute of Medical Research Israel-Canada, Faculty of Medicine, Hebrew University of Jerusalem, Jerusalem, Israel; ^4^ Department of Hematology, Shaare Zedek Medical Center and Faculty of Medicine, Hebrew University of Jerusalem, Jerusalem, Israel; ^5^ Clinical Endocrinology Laboratory, Shaare Zedek Medical Center, Jerusalem, Israel; ^6^ Department of Pediatrics, Pediatric Pulmonology Unit, Shaare Zedek Medical Center and Faculty of Medicine, Hebrew University of Jerusalem, Jerusalem, Israel; ^7^ Department of Pediatrics, Infectious Diseases Unit, Shaare Zedek Medical Center and Faculty of Medicine, Hebrew University of Jerusalem, Jerusalem, Israel; ^8^ Pediatric Department A and the Immunology Service, Jeffrey Modell Foundation Center, Edmond and Lily Safra Children’s Hospital, Tel-Hashomer Medical Center, Affiliated to the Sackler Faculty of Medicine, Tel Aviv University, Tel Aviv, Israel; ^9^ The Jeffrey Modell Foundation Israeli Network for Primary Immunodeficiency, New York, NY, United States; ^10^ Department of Pediatrics, Allergy and Clinical Immunology Unit, Shaare Zedek Medical Center, Faculty of Medicine, Hebrew University of Jerusalem, Jerusalem, Israel

**Keywords:** COVID-19, immunodeficiency, IVIg, antibodies, XLA

## Abstract

**Purpose:**

Patients with X-linked agammaglobulinemia (XLA) are characterized by humoral impairment and are routinely treated with intravenous immunoglobulin (IVIG). In this study, we aimed to investigate the presence of severe acute respiratory syndrome coronavirus 2 (SARS-CoV-2) antibodies in IVIG preparations harvested globally and evaluate the transfer of SARS-CoV-2 antibodies to the XLA patient.

**Methods:**

A single-center, prospective cohort study was conducted in the period of November 2020 to November 2022. Clinical and laboratory data, specifically, SARS-CoV-2 spike IgG levels from the serum of 115 IVIG preparations given to 5 XLA patient were collected. Concurrently, SARS-CoV-2 spike IgG levels from the serum of the 5 XLA was collected monthly.

**Results:**

Five XLA patients were evaluated within the study period. All were treated monthly with commercial IVIG preparations. A total of 115 IVIG treatments were given over the study period. The origin country and the date of IVIG harvesting was obtained for 111 (96%) of the treatments. Fifty-four IVIG preparations (49%) were harvested during the COVID-19 pandemic of which 76% were positive (>50AU/mL) for SARS-CoV-2 spike antibodies which were subsequently transmitted to the XLA patients in an approximate 10-fold reduction. SARS-CoV2 spike IgG was first detected in IVIG batches that completed their harvest date by September 2021. Positive products were harvested from origin countries with a documented prevalence over 2,000 per 100,000 population.

**Conclusion:**

As the prevalence of COVID-19 infections rises, detection of SARS-CoV-2 spike IgG in commercial IVIG products increases and is then transmitted to the patient. Future studies are needed to investigate the neutralizing capabilities of SARS-CoV-2 IgG and whether titer levels in IVIG remain consistent as the incidence of infection and vaccination rates in the population changes.

## 1 Introduction

The pandemic eruption of severe acute respiratory syndrome coronavirus 2 (SARS-CoV-2) gave rise to coronavirus disease 2019 (COVID-19) affecting millions of people worldwide (1). Major risk factors for severe disease include advanced age, obesity, high blood pressure, chronic kidney disease and diabetes (1). However, a significant proportion of severe illness occurs in the absence of these risk factors (2). With the rapid emergence of COVID-19 research, inborn errors of immunity were implicated in increasing the morbidity and mortality of COVID-19 (3–5). The major risk factor were defects in type I IFN, and a phenocopy with pre-existing neutralizing type I IFN autoantibodies, which together accounted for up to 25% of life threatening COVID-19 disease (6). The data regarding X-linked agammaglobulinemia (XLA), a humoral immunodeficiency, is more controversial, with some studies showing asymptomatic or mild disease with prompt recovery (7, 8) and others reporting an increased morbidity and mortality (9). While risk factors for severe COVID-19 within inborn errors of immunity were not too dissimilar from that seen in the general population, decreased lymphocyte counts and Ig levels were additionally noted risk factors (8, 10).

XLA is characterized by an impaired humoral response secondary to pathogenic variants of the *BTK* gene, which plays a key role in B-cell differentiation. Patients manifest with nearly absent B cells with severely decreased production of Ig, leading to recurrent and severe sinopulmonary infections (11). Treatment of XLA patients relies on intravenous immunoglobulins (IVIG) which significantly reduces the number of invasive bacterial infections and progression of lung disease (11). Severe viral infections with enteroviruses, a complication seen in XLA patient, has not significantly declined in the era of IVIG therapy (12). Additionally, studies looking at trough levels of various pathogens demonstrated non-protective anti measles antibodies (13). This highlights the limited value of IVIG in protection from severe viral infections.

However, IVIG at high doses has been reported to be efficacious in treatment of viral pneumonia during outbreaks with influenza, Middle East respiratory syndrome coronavirus (MERS-CoV), SARS and respiratory syncytial virus (RSV) (14). During the COVID-19 pandemic high dose IVIG and standard dosing were trialed with conflicting results including reduced mortality (15–17), no difference in mortality (18) and increased adverse events (19). These studies consisted of a broad heterogeneity of patients and lack of information on the composition of the IVIG, specifically the presence of high titer neutralizing SARS-CoV2 IgG antibodies, making it difficult to infer whether this therapeutic option would be efficacious in a specific population, such as those with underlying humoral defects, and with a specific immunoglobulin product.

At the beginning of 2021 studies showed the presence of anti-SARS-CoV2 IgG antibodies in IVIG products from countries with high prevalence of COVID-19. Anti-SARS-CoV2 IgG was present in IVIG products from Spain and the US (20) with up to 80% of IVIG batches positive for anti-SARS-CoV2 antibodies in Italy (21). This was followed by several neutralization studies on commercial lots demonstrating a positive correlation with detection and neutralization capabilities that increased temporally and exponentially (22, 23). One such study applied pharmacokinetic extrapolations suggesting that regularly treated PID patents may obtain a potential steady state trough level (23). This study also directly compared convalescent serum to IVIG products showing that 50% of plasma donation for convalescent serum lacked neutralizing titers, in contrast to IVIG in which there was lot to lot variability however neutralizing capacity means substantially increased with the passing of time (23).

Herein, we aimed to examine the presence and levels of anti- SARS-CoV-2 spike IgG antibodies in the different IVIG product harvested before and during the COVID-19 pandemic, along with the prevalence of COVID-19 in the origin of harvesting. Lastly, simultaneous measurements of SARS-CoV2 spike antibodies in the sera of XLA patients was collected to determine whether SARS-CoV2 spike antibodies are detected consistently and the relationship to COVID-19 prevalence in the origin of harvesting.

## 2 Methods

### 2.1 Study design and population

This is a prospective cohort study that was undertaken spanning over a two-year period (November 2020- November 2022) at the Allergy and Clinical Immunology Unit of Shaare Zedek Medical Center in Jerusalem, Israel. Five known XLA patients, with a diagnosis based on immune and or genetic workups, who were treated with IVIG were included in the study and followed monthly. At each encounter, total IgG and SARS-CoV-2 spike IgG antibodies were measured from the patients’ sera and from their IVIG preparation. Additional data collected at each monthly visit included reports of COVID19 infection, COVID-19 vaccination, and treatment with other anti-COVID therapies. Commercial IVIG products included Kedrion, Omerix, Grifols Flebogamma and Takeda Kiovig. Documentation of the harvesting date, country, and region of plasma harvesting was collected.

Secondary data collected included κ-deleting recombination excision circles (KREC) analysis, antibody levels at time of diagnosis, B cell percentages, review of their genetic testing for BTK deficiency, BTK protein detection by flow cytometry and co-morbidities associated with XLA, such as neutropenia, bronchiectasis, and other co-morbid conditions.

### 2.2 Genetic workup of XLA patients

XLA patients were diagnosed using whole exome sequencing (WES) and in one case using multiplex ligation-dependent probe amplification of the BTK gene. Functional confirmation of BTK variants of uncertain significance (VUS) were determined pathogenic *via* a combination of family segregation studies and BTK expression on monocytes and dendritic cells by using flow cytometry.

### 2.3 SARS-CoV-2 spike IgG assays

Testing of anti-SARS-CoV2 IgG antibodies was conducted using the Abbott AdviseDx SARS-CoV-2 IgG II assay according to the manufacturer’s instructions, as previously described (24). The ELISA kit is designed for detection of anti-SARS-CoV2 IgG against receptor binding domain (RBD) of the spike protein S1 of SARS-CoV-2. The Abbott AdviseDx SARS-CoV-2 IgG II assay calculates antibody concentrations expressed as arbitrary units (AU/mL) with >50AU/mL considered a positive result.

### 2.4 Immune analysis

Lymphocytes subsets were determined by flow-cytometry. KREC copies were determined using quantitative real-time polymerase chain reaction (RQ-PCR), as previously described (25), using 0.5-ug genomic DNA (gDNA) extracted from patients PBMCs. RQ-PCR was carried out using StepOne Plus Sequence Detector System (Applied Biosystems). A standard curve was constructed by using serial dilutions containing 10>3 and 10>6 copies of a plasmid with know KREC copy numbers. Patients’ samples were tested in duplicates, and the number of KRECs in each sample was calculated by comparing the obtained cycle threshold value of the sample to the standard curve using an absolute quantification algorithm. Amplification of RNAseP (TaqMan assay, Applied Biosystems) served as a quality control to verify similar amounts of genomic DNA that were used in the assay. When the KRECs termed signal joint (sj) are formed, an intron RSS-IGKDEL coding joint (cj) is simultaneously formed and is stably retained in the genomic DNA and thus can serve to monitor active B cell replication. Median sj-KREC in healthy controls is 447.9 copies/0.5 ug (range 86.2-2471.9 copies/0.5 ug DNA). Median (cj) in healthy control is 1061.1copies/0.5ugDNA (range 132-99989.9 copies/0.5ug). The ratio between the sj-KREC copies and the (cj) copies measured represents the homeostasis of both B cell neo-genesis and the replication history of B lymphocyte subsets in our BTK deficient patients.

### 2.5 Ethical review of the study

The study was approved by the institutional review board (IRB) committee of Shaare Zedek Medical Center, Jerusalem, Israel (IRB number: SZMC-20-0525). Patients were given explanations regarding the study and signed informed consents.

### 2.6 Statistical analysis

Data was collated using Microsoft Excel™ 365. Statistical analysis was done using Prism GraphPad 9.3.1. (GraphPad Software, San Diego, California USA, www.graphpad.com).

## 3 Results

### 3.1 Clinical characteristics of the patients

During a period from November 2020 to November 2022, five XLA patients (P1-5) were followed prospectively, monthly, with routine IVIG replacement therapy (**Table 1**). Five male patients, age range of 2-37 years were included. Four out of the five patients were children and four were of Jewish ancestry. All were compliant on long term therapy with IVIG. Three of the patients had bronchiectasis, four had transient neutropenia and none had comorbid conditions such as diabetes, kidney disease, obesity, or liver disease. None were on immunomodulatory therapies. One adult patient (P5) was vaccinated to COVID-19, none of the children were vaccinated to COVID-19. Three out of five patients (P1, 4 and 5) contracted COVID-19 during the study period with one patient (P5) having two separate infections and required hospitalization. P1 and P5 were diagnosed with COVID-19 based on a positive PCR. P4 had a sudden detectable SARS-CoV2-spike IgG titer, which was not seen in previous month, thus confirming COVID19 diagnosis. All patients recovered and no deaths occurred.

**Table 1:** Patient Demographics, Clinical Characteristics, Immune Evaluations and COVID19 history.

Patient	Age (years)	Ethnicity	IVIG infusions	CD19+ B-cell counts (% of lymphocytes, cells x109/L)	KREC	Ig at Diagnosis	BTK gene variants	BTK protein expression by flow cytometry	Comorbidities	COVID 19 Vaccination	COVID-19 Infection (date)
P1	9.2	Jewish	25	0.0(0)	UN	IgG UN, IgA <6.7, IgM <19	Ch X deletion of exons 2-5a	N/A	Bronchiectasis	No	1/2022
P2	4.11	Arabic	23	0.2 (13)	sj 22 (cj) 11.3	IgG <7, IgA <25.9, IgM <18	Ch X c.1076T>A Ile359Asn exon 12 missense pathogenic variants	Normal	Neutropenia	No	No
P3	12.7	Jewish	21	0.5 (200)	sj 26 (cj) 19	IgG 291, IgA<6.5 IgM <17 (age 2.5)	Ch X c.982C>T Gln328Tera	N/A	None	No	No
P4	10.6	Jewish	25	0.3 (18)	sj 74.7 (cj)51.4	IgG 974, IgA <6.2, IgM <18)	Ch X exon 2 His28Arg	N/A	Bronchiectasis Neutropenia	No	10/2021
P5	39	Jewish	21	0.0 (0)	UN	IgG UN, IgA<6.1, IgM <17	N/A	N/A	Bronchiectasis	Yes	9/2020 3/2021 9/2021

XLA, X-linked agammaglobulinemia; IVIG, Intravenous immunoglobulins; KREC, κ-deleting recombination excision circles; Ig, Immunoglobulins; N/A, Data is not available; UN, undetectable. a – novel variants.

### 3.2 B cell number and functional assays including KREC analysis of the XLA patients

Immune workup of the patients is summarized in **Table 1**. P1 and P5 had 0% CD19+ B cells with absent IgG, IgA, and IgM at the time of diagnosis. KREC analysis during the study period showed undetectable KRECs.

P2 had 0.2% CD19+ B cells and undetectable IgG, IgA and IgM at the time of diagnosis. Functional testing for BTK showed expression of BTK on dendritic cells and on the small number of present B cells. KREC analysis during the study period showed nearly absent detection of B cell rearrangement with sj-KRECs of 22 (reference range 86.2-2471.9 copies/0.5 ug DNA) and (cj) 11.3 (reference range 132-99989.9 copies/0.5ug).

P3 had 0.5% CD19+ B cells at the time of diagnosis and a detectable IgG of 291 (>2SD below normal for age) with undetectable IgA and IgM. KREC analysis during the study period similar to P2 showed an sj-KREC of 26 (reference range 86.2-2471.9 copies/0.5 ug DNA) and (cj) 19 (range 132-99989.9 copies/0.5ug). P2 and P3 only had SARS-CoV2-spike IgG present when receiving anti-SARS-CoV2-IVIG.

P4 had 0.3% CD19 expressing B cells at the time of diagnosis at age 3. Inconsistent with typical XLA he had a normal IgG count with absent IgA and IgM. Specific antibody results were not attainable. KREC analysis during the study period showed a higher presence of receptor rearrangement compared to the other XLA patients and was significant for sj-KREC of 74.7 (range 86.2-2471.9 copies/0.5 ug DNA) and (cj) 51.4 (range 132-99989.9 copies/0.5ug) (**Table 1**). P4 was the only patient that had presence of SARS-CoV2-spike IgG prior to receiving anti-SARS-CoV2-IVIG, suggesting an ability to mount his own titer.

### 3.3 Genetic testing and functional validation

Four out of the five patients completed genetic testing of the BTK gene of which three were sequencing analysis and one was multiplex ligation-dependent probe amplification of the *BTK* gene. Three of the patients have novel variants, that were determined pathogenic, one had a known pathogenic variant with conflicting reports on severity of this variants. Lastly one patient had missing genetic analysis.

P1 has a family history of an uncle with XLA, prompting testing soon after birth inclusive of multiplex ligation-dependent probe amplification of the *BTK* gene which identified a novel deletion of exons 2-5. WES was not completed. The X-linked inheritance pattern in P1’s family along with immune studies confirming 0% B cells with undetectable Ig and absent KREC confirm the variant’s pathogenicity. In line with reports studying genotype-phenotype correlations the lack of B cells, low IgM and absent KREC are consistent with a classic early onset XLA (26).

P2 presented with neutropenia and *Haemophilus Influenza* pneumonia by age two, prompting an immunologic and genetic evaluation. There was no family history of immunodeficiency, and he has one healthy brother. WES revealed a novel and rare missense variant, frequency of zero, in exon 12, c.1076T>A (p. Ile359Asn), determined as likely pathogenic. BTK expression was present on dendritic cells and on 0.2% of B cells present. Functional testing of B cells *via* KREC analysis of the present B cells showed a significantly depressed to nearly absent sj and (cj). Taken together, although there is presence of BTK, the function is impaired and consistent with a diagnosis of XLA.

P3 was tested and diagnosed soon after birth given a known family history of XLA. WES revealed a novel nonsense variant c.982C>T (p.Gln328Ter). This variant has been previously reported in another XLA patient (27). Functional testing of B cells *via* KREC analysis showed a significantly depressed to nearly absent sj-KREC and (cj). Taken together his nearly absent KREC, low B cells, severely decreased Ig and X linked inheritance pattern confirms pathogenicity of the p.Gln328Ter nonsense variant.

P4 presented at the age of 4 years with multiple infections including pneumonia, empyema, pneumococcal septic arthritis, and bacteremia leading to an immunodeficiency and genetic evaluation. He was noted to have normal IgG with absent IgA and IgM. Lymphocyte enumeration revealed 0.3% CD19 B cells. WES revealed a *de novo* pathogenic variant in exon 2 c.83G>A (p.Arg28His). This missense variant, located in exon two encoding the PH domain, leads to destruction or decrease in the protein IP4 binding capacity compared to wild type (28, 29). In a Chinese cohort of 174 XLA patients, Arg28His was the second most common variant (30) and was associated with a typical phenotype. Different amino acid substitutions at this site, such as R28C, lead to milder phenotypes in mice models (30) and in humans (30) with one described patient with a selective polysaccharide immunodeficiency (31, 32). Our patient presented at an older age of onset with presence of IgG, and low-level detection on KREC analysis. In summary Arg28His in our patient likely led to a semi-functional protein with a clear halt in B cell maturation as displayed by low B cell percentages with an initial ability for IgG production.

Of note, P5 was diagnosed with XLA more than 35 years ago, although he had no genetic confirmation of *BTK* variant. However, he had a brother with no B cells in peripheral blood and agammaglobulinemia, who died at a young age. Taken together with P5’s clinical presentation of infections starting at a young age, absent B cells, agammaglobulinemia, bronchiectasis and a family history of an affected male (**Table 1**), we included P5 as a probable XLA patient.

### 3.4 IVIG preparations, which are harvested in COVID-19 high-prevalence regions, contain SARS-CoV2 spike IgG

Data concerning IVIG preparations including the date and time of harvest were collected by reaching out to the associated pharmaceutical company. The IVIG obtained at Shaarei Zedek included Kedrion Ig-vena, Omerix Omr-IgG-am, Grifols Flebogamma, Grifols Gamnuex, Genmedix Privigen and Takeda Kiovig. Data was obtained for 111 (96%) of a total of 115 IVIG infusions given during the study period (**Table 2**). Reasons for missing data include technical difficulties. Majority of the IVIG products were harvested in the US with the remainder being harvested in Eastern Europe and Israel. 57 (51%) were harvested before the COVID-19 pandemic and 54 (49%) were harvested during the pandemic. Of the pandemic harvested IVIG, 13 of the preparations (24%) were negative for SARS-CoV2-spike IgG with the remainder 41 (84%) being positive for SARS-CoV2-spike IgG, inclusive of all four pharmaceutical preparations. A noted trend was a doubling in the SARS-CoV2-spike IgG from February 2021 to March 2021 despite a steady COVID-19 prevalence rate in Israel. This was the period of vaccine initiation, with world renowned high vaccination rates in Israel.

**Table 2:** IVIG preparation harvesting details and SARS-CoV-2 antibodies titer levels found in preparation. (COVID-10 prevalence from https://covid19.who.int/).

Commercial	Preparation amount	IVIG harvesting country	Last harvested (month. year)	COVID-19 prevalence (per 100,000 population)	COVID-19 antibodies (Au/ml, mean ± SD)
Kedrion	1	POLAND	7.20	124	20
Omrix	1	ISR	6.20	311	25
Omrix	1	ISR	7.20	774	10
Grifols	7	USA	6.20	840	13 ± 4
Kedrion	2	HUN GER USA	4.20	30+196+330	24.5 ± 0.7
Kiovig	10	USA+EUROPE	9.20	2192+911	117 ± 11
Omrix	1	ISR	2.21	8602	21
Omrix	1	USA	10.20	2732	117
Kiovig	9	USA+EUROPE	10.20	2732+1890	306 ± 21
Grifols	4	USA	12.21	4354	233 ± 10
Grifols	9	USA	1.21	8018	528 ± 57
Grifols	2	USA	3.21	9099	583 ± 2.8
Omrix	1	ISA	3.21	8967	612
Tekada	1	USA+ EUROPE	10.21	13531+10321	792
Genmedix	4	N/A	2.23	N/A	>40K

N/A, Data is not available.

IVIG preparations containing SARS-CoV2 spike IgG (anti-SARS-CoV2-IVIG), were all harvested from regions of high COVID-19 prevalence COVID-19 (>2,000 per 100,000 population) while the negative preparations were harvested from regions with lower COVID-19 prevalence (**Table 2**).

### 3.5 SARS-CoV-2 antibodies of XLA patients after receiving IVIG preparations positive for SARS-CoV2 spike antibody

During the study period, five patients were treated 115 times, of which 54 IVIG contained anti-SARS-CoV2-IVIG.

**Figure 1:**
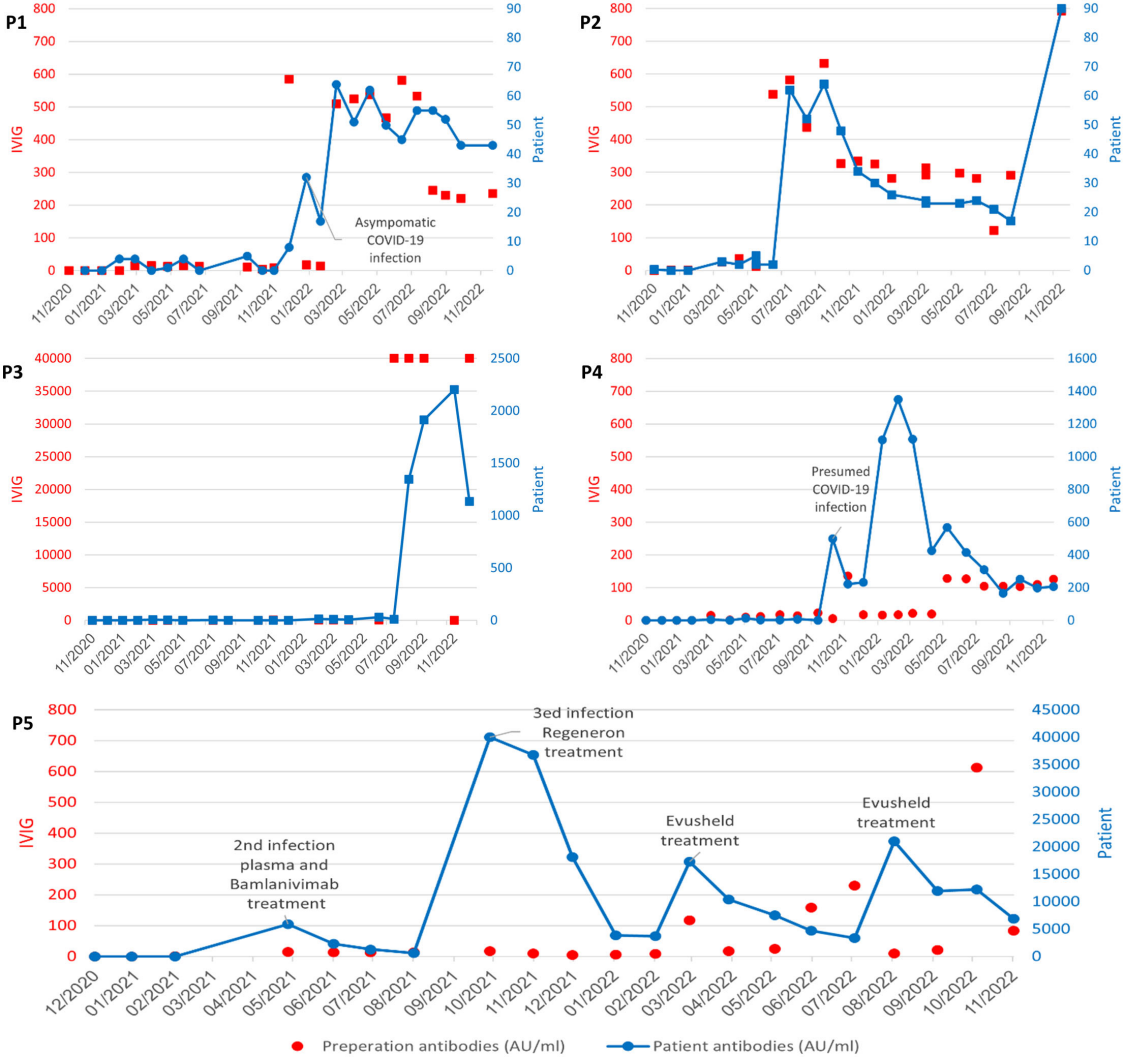
Titer levels reported in AU/mL in commercial IVIG products and patient serum A-P1; B- P2; C-P3; D- P4; E-P5. SARS-CoV2 antibody levels in IVIG preparations (left y axis, red) with reflected patient serum SARS-CoV2 antibody levels over time (right y axis, blue). P1, P2 and P3 treated with high and low titer SARS-CoV2 IVIG (red)with reflective rise and fall in mean patient SARS-CoV2 antibody levels (blue) over time. P4: Detection of SARS-CoV2 antibody levels (499AU/mL) in P4 noted prior to receiving SARS-CoV2 IVIG suggestive of asymptomatic infection. This was followed by low titer SARS-CoV2 IVIG and lower patient serum titers. P5: high titer patient SARS-CoV2 antibody levels reflective of treatment with Bamlanivimab, Casirivimab/imdevimab (Regeneron) and Tixagevimab/cilgavimab (Evusheld).

P1 received over 10 IVIG treatments lacking SARS-CoV2-spike IgG, which was reflected in the patient’s serum (mean of 1AU/mL). He was then treated with IVIG containing a high level of anti-SARS-CoV2-IVIG (585 AU/ml) followed by a first time detection of SARS-CoV2-spike IgG in the patient’s serum the following month (32AU/ml). At that time, he was found to be PCR positive to COVID-19 without any symptomatology (**Figure 1**). The following six IVIG treatments containeing anti-SARS-CoV2, three were harvested from high prevalence regions and three were harvested from low prevalence regions. His serum reflected a mean persistently detectable SARS-CoV2-spike IgG (54 Au/mL) while treated with the IVIG from high prevalence regions (mean 525 AU/mL) and dropped (39 Au/mL) while treated with IVIG from lower prevalence regions (229 Au/mL) (**Figure 1**).

P2 had undetectable SARS-CoV2-spike IgG while on IVIG lacking SARS-CoV2-spike IgG. He was then treated multiple times with IVIG harvested from a high prevalence region (SARS-CoV2-spike IgG mean 547 AU/ml) and was found to have a mean serum SARS-CoV2-spike IgG levels of 56 AU/ml for the following months. Subsequently he was infused with IVIG harvested from a lower prevalence region (SARS-CoV2-spike IgG mean = 304 AU/ml) with negative SARS-CoV2-spike IgG levels in the patient’s’ serum, in the following months (mean = 25 AU/ml) (**Figure 1**). P2 did not have a history of COVID-19 infection during the study period.

P3 was treated with IVIG lacking anti-SARS-CoV2 antibodies during most of the study period, reflected in undetectable SARS-CoV2-spike IgG in his serum. He was then treated with an IVIG containig an extremely high titer of anti-SARS-CoV2 (40,000 Au/mL) with a reciprocal spike of SARS-CoV2-spike IgG detected in the patients serum (2202 AU/ml). His next IVIG infusion was without SARS-CoV2-spike IgG and the patient serum SARS-CoV2-spike IgG dropped by 50% (1,136 AU/mL) (**Figure 1**). P3 has a reported negative COVID-19 infection history with no document positive PCR.

P4 had a abrupt detectable SARS-CoV2-spike IgG (increased from 1 AU/mL to 499AU/mL in one month) prior to receiving any anti-SARS-CoV2 containing IVIG, suggestive of an asymptomatic infection and an unexpected ability to make specific antibodies. The patient then received over ten IVIG infusions harvested from lower prevalence regions or IVIG lacking SARS-CoV2-spike IgG. The patient continued to have detectable SARS-CoV2 spike antibodies in his serum, with titers of up to 1351 Au/mL (**Figure 1**).

**Figure 2:**
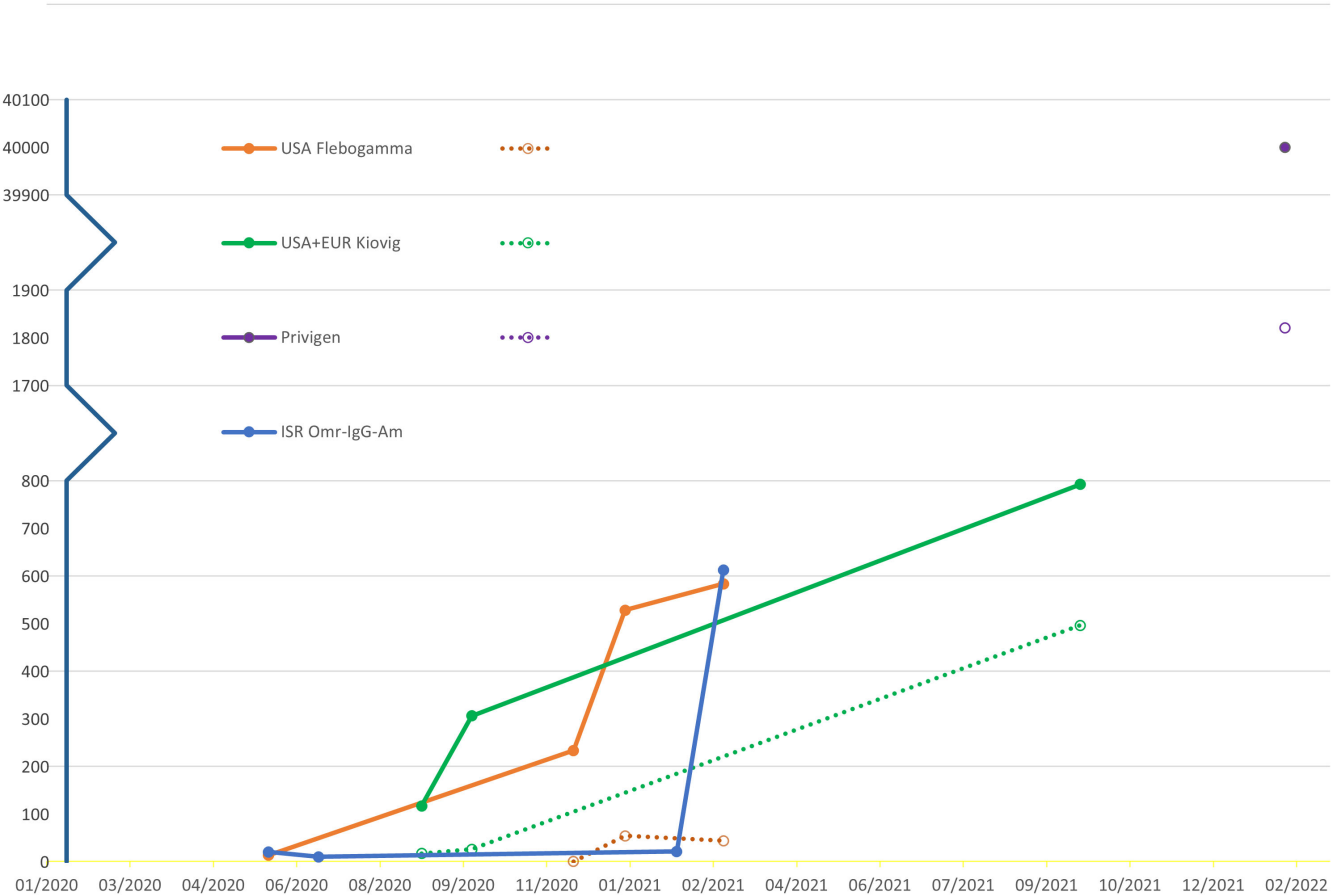
Average SARS-CoV2-spike antibody titer levels measured in AU/mL over time Different commercial products (solid lines), XLA patients’ sera (dotted lines).

Overall, SARS-CoV2 spike antibodies greater than 50AU/mL SARS-CoV2 in our patients’ sera was consistently detected in those treated with IVIG preparations containing anti-SARS-CoV2-levels above 500AU/mL. These anti-SARS-CoV2 IVIG preparation were harvested from populations with COVID-19 prevalence of approximately 8,000 per 100,000 population. Additionally, with the passing of time, we found an elevation of the average titer level in the commercial IVIG products which was reflected in the patients’ sera (**Figure 2**).

### 3.6 Recurrent infections in an XLA patient and SARS-CoV-2 IgG titers following several treatments

P5, the only adult XLA patient in the study, received three infusions with IVIG from the pre-pandemic period, with undetected SARS-CoV2-spike IgG in his serum. He was infected three times with COVID-19 (one prior to the study period). His second infection was treated with hyperimmune SARS-CoV2 Ig and bamlanivimab with the patient having a peak antibody level of 5,800 AU/mL with continued detection for four months. Hence he had his third COVID-19 infection and was treated with Casirivimab/imdevimab (Regeneron) with a peak antibody level of 40,000 AU/mL, with continued detection five months later as high as 3,600 AU/mL. The patient was then treated twice with Tixagevimab/cilgavimab (Evusheld) as prophylaxis with peak SARS-CoV2 Ig levels in his sera of 17,000AU/mL and 21,000AU/mL respectively. At the end point of the study, four months post- Evusheld SARS-CoV2 Ig antibody levels in his sera continued to be high (5,800AU/mL).

### 3.7 SARS-CoV-2 antibodies of XLA patients after vaccinations

Four patients were of pediatric age and did not receive SARS-CoV2 vaccination during the study period. P5, the only adult patient was vaccinated to SARS-CoV2. Spike antibodies were detected in his serum only while on treatment with anti-SARS-CoV2-IVIG, convalescent plasma and Evusheld and therefore response to vaccination were unable to be determined.

## 4 Discussion

In this study, we analyzed commercial IVIG from four different companies for SARS-CoV-2 antibody concentrations in the period of November 2020 to November 2022. Our study has several limitations including unknown regions of harvesting within each country with known pockets of higher prevalence and outbreaks of infections which can impact the ratio of prevalence to titer levels. Additionally, exact dates of harvesting are unknown with commercial companies reporting a block of months from which the product was harvested. SARS-CoV2-spike IgG was present in 76% of IVIG products that were harvested during the COVD-19 pandemic. Detection of SARS-CoV2-spike IgG in the commercial products was seen at a prevalence of greater than approximately 2,000 per 100,000 population.

Detection of SARS-CoV-2 antibodies in the patients’ sera was seen while receiving IVIG harvested from high prevalence regions (greater than approximately 8,000 per 100,000) with a titer level of above 500 AU/mL in the IVIG preparation. Additionally, as more consistent infusions with high prevalence lots were given (nearing 500 Au/ML) there was more consistent presence of SARS-CoV2-spike IgG in the patients. Conversely, with repeated infusions from lower prevalence lots (150-300Au/mL range) the patients serum SARS-CoV2-spike IgG dropped to below 50Au/ML. Trends that were noted (as seen in P1 and P2) was a mirroring of the level in the patient’s sera to what was present in the IVIG with an approximated 10-fold reduction level from IVIG to the patient’s sera.

Additionally, with the passage of time, regardless of prevalence rate, there was a mean SARS-CoV2-spike IgG rise in IVIG products and in the patients’ sera. This is likely explained by the increase in vaccination rates and a higher percentage of the population with a positive COVID-19 infection history. Of note, in commercial lots harvested in Israel there was doubling in the SARS-CoV2-spike IgG in harvested IVIG from February 2021 to March 2021 despite a steady COVID-19 prevalence rate in Israel. This was the period of vaccine initiation with world renowned high vaccination rates in Israel. This finding demonstrates that presence of SARS-CoV2-spike IgG is likely dependent on both prevalence rates but also vaccination rates.

Our study was unable to identify the clinical benefits of anti-SARS-CoV2-IVIG, as we were unable to report COVID-19 infection in two of the five patients. However, of the three patients with known COVID-19 illness, two had an asymptomatic course and did not have previously reported risk factors for severe COVID-19 disease. The only symptomatic COVID-19 infection was seen in P5, the oldest patient in our cohort with risk factors associated with a more complicated course, such as lung disease, absent B cells, undetectable Ig and absent KREC analysis (7–10). Moreover, products, such as Evusheld, have higher titer levels of SARS-CoV2 spike IgG than what is seen in commercial IVIG or convalescent plasma. This was translated into higher titer levels in the patients’ sera of SARS-CoV2 spike Ig that remained elevated for up to 4 months post- treatment. This highlights the use of more reliable targeted monoclonal therapies leading to higher titers, longer half-lives and non-dependence on the population’s infection and vaccination prevalence.

Of note, P2 has a novel and extremely rare *BTK* likely pathogenic variant. However, BTK expression on flow cytometry in this patient was normal. This does not rule out the diagnosis, as there are cases of normal BTK expression with aberrant protein function (33). Available assays for functional validation included quantification of B-cell number, immunoglobulin levels, KREC analysis. P2 has a lack of B cells, undetectable immunoglobulins, and lack of KREC. In addition, the *BTK* variant is extremely rare and was reported as likely pathogenic by different prediction software. Searching the online BTK database (34), reported is a pathogenic variant at the same site, although to a different amino acid of c.1076T>G, p.Ile359Ser. Our patient was c.1076T>A, p.Ile359Asn. Lastly, no other genes were reported in his WES that could better explain his disease. Taken together. we believe that this is consistent with a diagnosis of XLA.

KREC analysis was completed in all 5 XLA patients to assess if hypomorphic XLA may have a milder course or an ability to mount antibody responses to natural infection and or vaccination. Of the five patients, two had undetectable KRECs and three had KRECS just above detection level. Only one of the patients, P4, with detectable level of KRECs and peripheral B cells, was able to mount SARS-CoV2-spike IgG. P4 is also the only patient with a BTK pathogenic variant that has been associated in some cases with B cell lymphopenia with normal immunoglobulin levels and a selective polysaccharide immunodeficiency.

In conclusion, IVIG preparations from high-prevalence COVID-19 regions contain higher detectable SARS-CoV-2 IgG antibodies than those from lower prevalence regions. Detection of SARS-CoV2 spike antibody >50AU/ml in patients’ sera was only seen in those treated with IVIG from high prevalence regions >8,000 per 100,0000 population, with mean serum levels of approximately 500 AU/mL. Future studies assessing whether global vaccination rates and broad harvesting can achieve an expected steady state level of SARS-CoV2-spike IgG in commercial lots that will be less dependent on prevalence rates of natural infection can be expected.

## Data availability statement

The raw data supporting the conclusions of this article will be made available by the authors, without undue reservation.

## Ethics statement

The study was approved by the institutional review board (IRB) committee of Shaare Zedek Medical Center, Jerusalem, Israel (IRB number: SZMC-20-0525). Written informed consent to participate in this study was provided by the participants’ legal guardian/next of kin. Written informed consent was obtained from the individual(s), and minor(s)’ legal guardian/next of kin, for the publication of any potentially identifiable images or data included in this article.

## Author contributions

AR- data collection, analysis and writing the first draft; OS-data analysis writing of the first draft and manuscript revisions; RE- data analysis and writing of manuscript; AT- treatment of patients; SM, laboratory workup; SG, OM- treatment of patients; AL – immune workup; AS- genetic and immune workup; YT, RS- Immunological consultation; OT- treatment of patients, study conceptualization, data analysis, manuscript revisions and supervision. All authors contributed to the article and approved the submitted version.

## References

[1] Rothan HA Byrareddy SN. The epidemiology and pathogenesis of coronavirus disease (COVID-19) outbreak. J Autoimmun (2020) 109:102433. doi: 10.1016/j.jaut.2020.102433 32113704 PMC7127067

[2] Callender LA Curran M Bates SM Mairesse M Weigandt J Betts CJ. The impact of pre-existing comorbidities and therapeutic interventions on COVID-19. Front Immunol (2020) 11:1991. doi: 10.3389/fimmu.2020.01991 32903476 PMC7437504

[3] Zhang Q Bastard P Liu Z Le Pen J Moncada-Velez M Chen J. Inborn errors of type I IFN immunity in patients with life-threatening COVID-19. Science (2020) 370(6515). doi: 10.1126/science.abd4570 PMC7857407 32972995

[4] Fallerini C Daga S Mantovani S Benetti E Picchiotti N Francisci D. Association of toll-like receptor 7 variants with life-threatening COVID-19 disease in males: findings from a nested case-control study. Elife (2021) 10. doi: 10.7554/eLife.67569 PMC7987337 33650967

[5] Asano T Boisson B Onodi F Matuozzo D Moncada-Velez M Maglorius Renkilaraj MRL. X-Linked recessive TLR7 deficiency in ~1% of men under 60 years old with life-threatening COVID-19. Sci Immunol (2021) 6(62). doi: 10.1126/sciimmunol.abl4348 PMC8532080 34413140

[6] Liu W Xu ZQ Long YJ Feng MQ. Replenishment of urban landscape ponds with reclaimed water: Spatiotemporal variations of water quality and mechanism of algal inhibition with alum sludge. Sci Total Environ (2021) 790:148052. doi: 10.1016/j.scitotenv.2021.148052 34090163

[7] Meyts I Bucciol G Quinti I Neven B Fischer A Seoane E. Coronavirus disease 2019 in patients with inborn errors of immunity: An international study. J Allergy Clin Immunol (2021) 147(2):520–31. doi: 10.1016/j.jaci.2020.09.010 PMC7832563 32980424

[8] Tangye SG Bucciol G Meyts I. Mechanisms underlying host defense and disease pathology in response to severe acute respiratory syndrome (SARS)-CoV2 infection: insights from inborn errors of immunity. Curr Opin Allergy Clin Immunol (2021) 21(6):515–24. doi: 10.1097/ACI.0000000000000786 34494617

[9] Delmonte OM Castagnoli R Notarangelo LD. COVID-19 and inborn errors of immunity. Physiol (Bethesda) (2022) 37(6):0. doi: 10.1152/physiol.00016.2022 PMC9550578 35944006

[10] Kuster JK Unlu S Makin TA Par-Young J Simonov M Shafi S. Low IgG trough and lymphocyte subset counts are associated with hospitalization for COVID-19 in patients with primary antibody deficiency. J Allergy Clin Immunol Pract (2022) 10(2):633–6.e3. doi: 10.1016/j.jaip.2021.11.030 34929372 PMC8683251

[11] Shillitoe BMJ Gennery AR. An update on X-linked agammaglobulinaemia: clinical manifestations and management. Curr Opin Allergy Clin Immunol (2019) 19(6):571–7. doi: 10.1097/ACI.0000000000000584 31464718

[12] El-Sayed ZA Abramova I Aldave JC Al-Herz W Bezrodnik L Boukari R. X-Linked agammaglobulinemia (XLA):Phenotype, diagnosis, and therapeutic challenges around the world. World Allergy Organ J (2019) 12(3):100018. doi: 10.1016/j.waojou.2019.100018 30937141 PMC6439403

[13] Hassin O Abu Freih Y Hazan R Lev A Zrihen KS Somech R. Trough concentrations of specific antibodies in primary immunodeficiency patients receiving intravenous immunoglobulin replacement therapy. J Clin Med (2021) 10(4). doi: 10.3390/jcm10040592 PMC7915625 33557365

[14] Liu X Cao W Li T. High-dose intravenous immunoglobulins in the treatment of severe acute viral pneumonia: The known mechanisms and clinical effects. Front Immunol (2020) 11:1660. doi: 10.3389/fimmu.2020.01660 32760407 PMC7372093

[15] Herth FJF Sakoulas G Haddad F. Use of intravenous immunoglobulin (Prevagen or octagam) for the treatment of COVID-19: Retrospective case series. Respiration (2020) 99(12):1145–53. doi: 10.1159/000511376 PMC7801971 33316806

[16] Gharebaghi N Nejadrahim R Mousavi SJ Sadat-Ebrahimi SR Hajizadeh R. The use of intravenous immunoglobulin gamma for the treatment of severe coronavirus disease 2019: a randomized placebo-controlled double-blind clinical trial. BMC Infect Dis (2020) 20(1):786. doi: 10.1186/s12879-020-05507-4 33087047 PMC7576972

[17] Shao Z Feng Y Zhong L Xie Q Lei M Liu Z. Clinical efficacy of intravenous immunoglobulin therapy in critical ill patients with COVID-19: a multicenter retrospective cohort study. Clin Transl Immunol (2020) 9(10):e1192. doi: 10.1002/cti2.1192 PMC7557105 33082954

[18] Tabarsi P Barati S Jamaati H Haseli S Marjani M Moniri A. Evaluating the effects of intravenous immunoglobulin (IVIg) on the management of severe COVID-19 cases: A randomized controlled trial. Int Immunopharmacol (2021) 90:107205. doi: 10.1016/j.intimp.2020.107205 33214093 PMC7665876

[19] Mazeraud A Jamme M Mancusi RL Latroche C Megarbane B Siami S. Intravenous immunoglobulins in patients with COVID-19-associated moderate-to-severe acute respiratory distress syndrome (ICAR): multicentre, double-blind, placebo-controlled, phase 3 trial. Lancet Respir Med (2022) 10(2):158–66. doi: 10.1016/S2213-2600(21)00440-9 PMC8585489 34774185

[20] Romero C Diez JM Gajardo R. Anti-SARS-CoV-2 antibodies in healthy donor plasma pools and IVIG products. Lancet Infect Dis (2021) 21(6):765–6. doi: 10.1016/S1473-3099(21)00059-1 PMC7906732 33606999

[21] Pisani G Cristiano K Simeoni M Martina A Pati I Carocci A. Detection of antibodies against SARS-CoV-2 both in plasma pools for fractionation and in commercial intravenous immunoglobulins produced from plasma collected in Italy during the pandemic. Blood Transfus (2022) 20(3):198–205. doi: 10.2450/2021.0055-21 34059195 PMC9068351

[22] Miller AL Rider NL Pyles RB Judy B Xie X Shi PY. The arrival of SARS-CoV-2-neutralizing antibodies in a currently available commercial immunoglobulin. J Allergy Clin Immunol (2022) 149(6):1958–9. doi: 10.1016/j.jaci.2022.03.026 PMC9023086 35465974

[23] Volk A Covini-Souris C Kuehnel D De Mey C Romisch J Schmidt T. SARS-CoV-2 neutralization in convalescent plasma and commercial lots of plasma-derived immunoglobulin. BioDrugs (2022) 36(1):41–53. doi: 10.1007/s40259-021-00511-9 34843105 PMC8628143

[24] Maine GN Krishnan SM Walewski K Trueman J Sykes E Sun Q. Clinical and analytical evaluation of the Abbott AdviseDx quantitative SARS-CoV-2 IgG assay and comparison with two other serological tests. J Immunol Methods (2022) 503:113243. doi: 10.1016/j.jim.2022.113243 35181288 PMC8847080

[25] Toker O Broides A Lev A Simon AJ Megged O Shamriz O. B cell repertoire in patients with a novel BTK mutation: expanding the spectrum of atypical X-linked agammaglobulinemia. Immunol Res (2022) 70(2):216–23. doi: 10.1007/s12026-022-09263-2 35001352

[26] Broides A Yang W Conley ME. Genotype/phenotype correlations in X-linked agammaglobulinemia. Clin Immunol (2006) 118(2-3):195–200. doi: 10.1016/j.clim.2005.10.007 16297664

[27] Chen XF Wang WF Zhang YD Zhao W Wu J Chen TX. Clinical characteristics and genetic profiles of 174 patients with X-linked agammaglobulinemia: Report from shanghai, China (2000-2015). Med (Baltimore) (2016) 95(32):e4544. doi: 10.1097/MD.0000000000004544 PMC4985333 27512878

[28] Fukuda M Kojima T Kabayama H Mikoshiba K. Mutation of the pleckstrin homology domain of bruton's tyrosine kinase in immunodeficiency impaired inositol 1,3,4,5-tetrakisphosphate binding capacity. J Biol Chem (1996) 271(48):30303–6. doi: 10.1074/jbc.271.48.30303 8939985

[29] Rawlings DJ Witte ON. The btk subfamily of cytoplasmic tyrosine kinases: structure, regulation and function. Semin Immunol (1995) 7(4):237–46. doi: 10.1006/smim.1995.0028 8520028

[30] Kojima T Fukuda M Watanabe Y Hamazato F Mikoshiba K. Characterization of the pleckstrin homology domain of btk as an inositol polyphosphate and phosphoinositide binding domain. Biochem Biophys Res Commun (1997) 236(2):333–9. doi: 10.1006/bbrc.1997.6947 9240435

[31] Ohta Y Haire RN Litman RT Fu SM Nelson RP Kratz J. Genomic organization and structure of bruton agammaglobulinemia tyrosine kinase: localization of mutations associated with varied clinical presentations and course in X chromosome-linked agammaglobulinemia. Proc Natl Acad Sci U S A (1994) 91(19):9062–6. doi: 10.1073/pnas.91.19.9062 PMC44747 8090769

[32] Wood PM Mayne A Joyce H Smith CI Granoff DM Kumararatne DS. A mutation in bruton's tyrosine kinase as a cause of selective anti-polysaccharide antibody deficiency. J Pediatr (2001) 139(1):148–51. doi: 10.1067/mpd.2001.115970 11445810

[33] Futatani T Miyawaki T Tsukada S Hashimoto S Kunikata T Arai S. Deficient expression of bruton's tyrosine kinase in monocytes from X-linked agammaglobulinemia as evaluated by a flow cytometric analysis and its clinical application to carrier detection. Blood (1998) 91(2):595–602. doi: 10.1182/blood.V91.2.595 9427714

[34] Databases for Immunodeficiency-Causing Variations. (2017). Available at: http://structure.bmc.lu.se/idbase/BTKbase/ [Accessed March 24, 2023].

